# Favored single nucleotide variants identified using whole genome Re-sequencing of Austrian and Chinese cattle breeds

**DOI:** 10.3389/fgene.2022.974787

**Published:** 2022-09-27

**Authors:** Maulana M. Naji, Yifan Jiang, Yuri T. Utsunomiya, Benjamin D. Rosen, Johann Sölkner, Chuduan Wang, Li Jiang, Qin Zhang, Yi Zhang, Xiangdong Ding, Gábor Mészáros

**Affiliations:** ^1^ University of Natural Resources and Life Sciences, Vienna, Austria; ^2^ China Agricultural University, Beijing, China; ^3^ Department of Production and Animal Health, School of Veterinary Medicine, São Paulo State University (Unesp), Araçatuba, Brazil; ^4^ Animal Genomics and Improvement Laboratory, USDA‐ARS, Beltsville, MD, United States

**Keywords:** cattle, whole-genome sequence (WGS), selection signature, *Bos taurus*, bos indicus, iSAFE, IHS, fst

## Abstract

Cattle have been essential for the development of human civilization since their first domestication few thousand years ago. Since then, they have spread across vast geographic areas following human activities. Throughout generations, the cattle genome has been shaped with detectable signals induced by various evolutionary processes, such as natural and human selection processes and demographic events. Identifying such signals, called selection signatures, is one of the primary goals of population genetics. Previous studies used various selection signature methods and normalized the outputs score using specific windows, in kbp or based on the number of SNPs, to identify the candidate regions. The recent method of iSAFE claimed for high accuracy in pinpointing the candidate SNPs. In this study, we analyzed whole-genome resequencing (WGS) data of ten individuals from Austrian Fleckvieh (*Bos taurus*) and fifty individuals from 14 Chinese indigenous breeds (*Bos taurus, Bos taurus indicus,* and admixed). Individual WGS reads were aligned to the cattle reference genome of ARS. UCD1.2 and subsequently undergone single nucleotide variants (SNVs) calling pipeline using GATK. Using these SNVs, we examined the population structure using principal component and admixture analysis. Then we refined selection signature candidates using the iSAFE program and compared it with the classical iHS approach. Additionally, we run Fst population differentiation from these two cattle groups. We found gradual changes of taurine in north China to admixed and indicine to the south. Based on the population structure and the number of individuals, we grouped samples to Fleckvieh, three Chinese taurines (Kazakh, Mongolian, Yanbian), admixed individuals (CHBI_Med), indicine individuals (CHBI_Low), and a combination of admixed and indicine (CHBI) for performing iSAFE and iHS tests. There were more significant SNVs identified using iSAFE than the iHS for the candidate of positive selection and more detectable signals in taurine than in indicine individuals. However, combining admixed and indicine individuals decreased the iSAFE signals. From both within-population tests, significant SNVs are linked to the olfactory receptors, production, reproduction, and temperament traits in taurine cattle, while heat and parasites tolerance in the admixed individuals. Fst test suggests similar patterns of population differentiation between Fleckvieh and three Chinese taurine breeds against CHBI. Nevertheless, there are genes shared only among the Chinese taurine, such as PAX5, affecting coat color, which might drive the differences between these yellowish coated breeds, and those in the greater Far East region.

## Introduction

Cattle are vital livestock for humans providing meat and milk for consumption, leather for protection, and power for plowing and transportation (FAO, 2015; Xu et al., 2015). Using available genetic evidence, there were two primary independent events postulated for the initial domestication of cattle, i.e., between 10,000 and 8,000 years ago for *Bos taurus* (*B. taurus*) in the Fertile Crescent and 8,000–6,000 years ago for *Bos taurus indicus* (*B. indicus*) in the Indus valley (Loftus et al., 1994). Since then, following human migration and trade, cattle have spread across the globe and undergone further evolutionary events for adaptation to local environments due to natural or selective breeding shaping each breed’s morphology, physiology, and behavior from its initial attributes (FAO, 2015; Xu et al., 2015; Wu et al., 2018). Currently, there are more than a thousand distinctive cattle breeds recognized worldwide (FAO, 2015).

The study of footprints in genes or genomics regions of livestock species due to the continuous evolutionary process is one of the main interests of population genetics (de Simoni Gouveia et al., 2014; Randhawa et al., 2016). With the development of genomics, these signals can be identified using single nucleotide polymorphisms (SNPs) arrays and whole-genome resequencing (WGS) data (Flori et al., 2009; Utsunomiya et al., 2013; Qanbari et al., 2014; Randhawa et al., 2014). These signals are inferred as they deviate from the neutral expectations in the patterns of genomic variations despite possible recombination events (Utsunomiya et al., 2013; de Simoni Gouveia et al., 2014). There are various proposed methods to detect these signals. Based on its approaches, they can be grouped into methods using local genetic diversity depression within a population, changes in allele frequency spectrum within and cross-populations, population allele differentiation across-populations, and haplotype homozygosity within and cross-populations (Utsunomiya et al., 2013; Randhawa et al., 2016).

Estimated for the first importation of *B. taurus* from West Asia around 3,900 years ago, there are ∼90 million cattle of various breeds in China, of which fifty-three of it are indigenous (Chen et al., 2018; National Bureau of Statistics, 2018). A previous study reported gradual transitions in cattle breed composition found across the country. *B. taurus is* predominantly found in the northern part, gradually admixed of *B. taurus* and *B. indicus* population in the central part, and pure *B. indicus* breeds to the southern part of the country (Chen et al., 2018). Another study (Zhang et al., 2020) using copy number variations (CNVs) supported that most Chinese breeds were hybrids of *B. taurus* and *B. indicus*.

Fleckvieh is a prominent dual-purpose breed in Austria with a population of around 1.5 million heads, corresponding to 76% of the total cattle population in the country (Kalcher et al., 2018). Also internationally known as Simmental, Fleckvieh genome was reported as one of the most studied *B. taurus* cattle after Frisian-Holstein (Randhawa et al., 2016). A previous study (Qanbari et al., 2014) utilized sequencing data of German Fleckvieh for selection signatures analysis. They employed ∼15 million autosomal SNPs inferred from the sequence data and found 106 candidates of selection regions linked to genes with the functionality of neuro-behavioral, sensory perception, and coat coloring patterns.

Most of the previous studies were limited in pinpointing exact locations of selection signatures, as they proposed large chunks of genomic regions in the size of a few kilobases to megabases as the candidates, containing many genes and thousands of polymorphisms (Randhawa et al., 2014; Xu et al., 2015; Bhati et al., 2020). They considered the region within linkage disequilibrium proximate as the candidate regions and reducing spurious effects of many SNPs signals as the reasons for using large scanning windows.

Recently developed methods of integrated Selection of Allele Favoured by Evolution - iSAFE are suggested to pinpoint the best candidate SNPs in selection signature regions (Akbari et al., 2018). iSAFE is designed to exploit signals from ongoing selective sweeps as it scores are based on the rank-order of the mutation in SNP candidates. Using phased genotype, this tool assigns intermediate score for each mutation based on the number of times it appears in different haplotypes weighted by total of all mutations found in the haplotype and its frequency. Then, overlapped scanning window is applied on these intermediate-scores of all mutations to find the best candidate SNP driving the selection, see Methods for details. iSAFE outperformed other tools, such as integrated Haplotype Score—iHS (Voight et al., 2006), in detecting favorable SNPs within large loci of 5 Mb without knowledge of demography, phenotype under selection, or functional annotations (Akbari et al., 2018). Thus, in this study, we aim to examine the candidate SNPs that drive the selection identified by iSAFE in genome-wide level, with no prior knowledge of candidate regions in selection, compared to the classical approach of integrated Haplotype Score—iHS (Voight et al., 2006; Szpiech and Hernandez, 2014) using sequence data of several Chinese breeds and Austrian Fleckvieh.

## Materials and methods

### Ethics statement

For this study, DNA was previously extracted from commercial AI bull semen straws. Thus, no ethical statement was further required.

### Alignment, variant calling, and phasing genotypes

In this study, we utilized whole genome re-sequencing of sixty individuals from fourteen Chinese and one Austrian cattle breeds, namely: Dabieshan (n = 2), Dehong (n = 2), Dengchuan (n = 2), Fujian (n = 2), Guanling (n = 2), Kazakh (n = 6), Liping (n = 2), Luxi (n = 2), Mongolian (n = 12), Nanyang (n = 2), Qinchuan (n = 2), Wenling (n = 2), Tibetan (n = 2), Yanbian (n = 10), and Fleckvieh (n = 10). In the analysis, we applied the alignment to SNV calling pipeline in China Agricultural University computational cluster for all Chinese breeds and Vienna Scientific Cluster for Austrian Fleckvieh.

BWA-mem v.0.7.17 (Li and Durbin, 2010) aligned paired-end reads of FASTQ against cattle reference genome ARS_UCD1.2 (Rosen et al., 2020), resulting in a sequence alignment map (SAM) file. Subsequently, samtools v.1.10 (Li et al., 2009) sorted SAM file by chromosomes and converted to binary alignment map (BAM). Picard (https://broadinstitute.github.io/picard/) functions of MarkDuplicates flagged duplicate reads in BAM files and function of AddOrReplaceReadGroups modified read groups information accordingly. For subsequent steps, GATK v.4.1 (McKenna et al., 2010) was used. GATK functions of BaseRecalibrator and ApplyBQSR detected and corrected base quality scores of mapped reads nearby known variants. GATK HaplotypeCaller with–ERC GVCF option called individual genotype for each BAM file.

Individual GVCFs files were combined using the GenomicsDB function of GATK, allowing combinations of GVCFs called using different versions of GATK. The joint cohort of GenotypeGVCFs called the final VCF file using parameter–allow-old-rms-mapping-quality-annotation-data since individual GVCFs were called using a different version of GATK in two different computational clusters. Subsequently, we retained single nucleotide variants using GATK SplitVcfs function and filter variants with the following parameters “QD < 2.0, QUAL <30, SOR >3, FS > 60, MQ < 40, MQRankSum <12.5, ReadPosRankSUm < -8.0” following general GATK’s recommendation.

We added ancestral alleles (Naji et al., 2021a) in the info column of the vcf file separately for each autosome using–fill-aa script of VCFTools v.0.1.15 (Danecek et al., 2011). Subsequently, Bcftools v.1.7 (Li et al., 2009) retained the biallelic SNPs in the VCF. Then, genotypes in the VCF file were phased using Beagle v.5.1 (Browning et al., 2018) and indexed using tabix v.1.7-2 (Li et al., 2009) resulting final phased data for the analysis.

### Principal component and admixture analysis

Before phasing steps, the multi-sample VCF file containing all autosomes was converted to binary plink format using VCFtools (Danecek et al., 2011). Plink1.9 (Chang et al., 2015) merged the dataset with additional individuals of Angus, Brahman, Gir, Holstein, Indian Zebu, Jersey, Kenana, Mangshi, Nelore, and Simmental breeds from the publicly available SRA NCBI database used in the previous study (Naji et al., 2021b). We filtered out variants with missing call rates exceeding 0.2. We used the–pca function with five eigenvectors for PCA on ∼4.5 million variants that were shared by all individuals. Admixture v.1.3 (Alexander et al., 2009) assessed population structures using the same input file as the PCA with K numbers of three to five. Outputs of PCA and admixture analysis were plotted using R (R Core Team, 2020).

### Scanning for SNVs driving positive selection

The iSAFE test ranks all SNVs within linkage-disequilibrium (LD) regions with selective sweep signals based on their contribution to the selection signal. The program (Akbari et al., 2018) scans for signals up to 5 Mb using statistics derived solely from haplotypes and ancestral allele information. Under the hood, iSAFE used two steps; first, it searched for the best candidate mutations using selection of allele favored by evolution (SAFE) and then combined those signals for the final iSAFE score for the maximum region spanning 5 Mb.

In the first step, haplotype allele frequency (HAF) is used to distinguish haplotypes based on the sum of derived allele counts. Haplotypes are considered ‘distinct’ once they have different HAF scores and ‘carries a mutation *e*' if they have derived allele at site *e* with *f* mutation frequency. When a particular haplotype is a putative carrier of a favored allele, its HAF score increases due to carrying more derived alleles.

$$k\left(e\right)=\frac{number\;of\;distinct\;haplotypes\;carrying\;mutation\;e}{number\;of\;distinct\;haplotypes\;in\;sample}$$

k(e) denotes a fraction of distinct haplotypes carrying mutation e, while ϕ(e) denotes the normalized sum of HAF scores carrying the mutation e.

$$\phi \left(e\right)=\frac{sum\;of\;HAF\;scores\;of\;haplotypes\;carrying\;mutation\;e}{sum\;of\;HAF\;scores\;of\;all\;haplotypes}$$

Based on these calculations, SAFE-score is defined as

$$SAFE\left(e\right)=\frac{\phi -k}{\sqrt{f\left(1-f\right)}}$$

Theoretically, selective sweeps will reduce the k(e) score as the number of distinct haplotypes carrying favored mutations is reduced. Increasing HAF scores in carrier haplotypes will reduce the ratio of total HAF-score contributed by non-carrier haplotypes, consequently higher ϕ value. Thus, the mutation with the highest SAFE score is expected as a candidate of favored mutation.

As the *k* score reduces its power to pinpoint favored mutation due to most haplotypes becoming unique in larger windows, thus it applies a set of half-overlapped windows (W) with a fixed size of 300 SNPs on the second step. δ denotes a list of selected mutations *e* in each window with the highest SAFE score. For mutation *e* in window w , $\psi_{e,w^{\prime }}$ denotes the larger SAFE score of e and 0 when e is inserted into window $w^{\prime }$ . $\psi_{e,w^{\prime }}$ will be relatively high when e is a favored mutation and the genealogies of *w* and $w^{\prime }$ are very similar. α(w) denotes the weight of each window w which would have a high value corresponding to favored mutations contained. The iSAFE score for mutation *e* is calculated by the higher SAFE score of *e* and weight of all scanning windows.

$$\alpha \left(w\right)=\frac{\sum_{e\in \delta }\psi_{e,w}}{\sum_{w^{\prime }\in W}\sum_{e\in \delta }\psi_{e,w^{\prime }}}$$

$$iSAFE\left(e\right)=\sum_{w\in W}\psi_{e,w}\;.\;\alpha \left(w\right)$$

We used the built-in program to pinpoint favored mutations for a non-overlapped window of 4 Mb in autosomes for each pool of individuals. Then, we concatenated iSAFE scores for all SNPs of all autosomes and applied the normal distribution’s right-tail probability density function (PDF) to infer the *p*-values as provided in R (R Core Team, 2020).

The iHS test was first proposed in 2006 and used for many studies to identify positive selections in livestock populations (Qanbari et al., 2014; Randhawa et al., 2016; Vatsiou et al., 2016). We used selscan (Szpiech and Hernandez, 2014) to perform the iHS test using phased data for each chromosome of individual pools. In the notation below, for each queried SNV $\left(x_{i}\right)$ , integrated haplotype homozygosity (iHH) of ancestral (0) and derived 1) haplotypes (C: = {0,1}) was calculated from the extended haplotype homozygosity (EHH) (Sabeti et al., 2002) from both upstream (U) and downstream (D) set of markers of each query site $\left(x_{i}\right)$ . $g\left(x_{i-1},\;x_{i}\right)$ represents the genetic distance between two markers created with an arbitrary value of one centiMorgan per megabase.

$${iHH}_{c}=\overset{\left|D\right|}{\underset{i=1}{\sum }}\frac{1}{2}\left({EHH}_{c}\left(x_{i-1}\right)+{EHH}_{c}\left(x_{i}\right)\right)g\left(x_{i-1},x_{i}\right)+\overset{\left|U\right|}{\underset{i=1}{\sum }}\frac{1}{2}\left({EHH}_{c}\left(x_{i-1}\right)+{EHH}_{c}\left(x_{i}\right)\right)g\left(x_{i-1},x_{i}\right)$$

The unstandardized iHS score was calculated as $iHS=\ln\frac{iHH1}{iHH0}$ where a positive value indicates unusual long haplotypes carrying derived alleles compared to the neutral model indicating recent positive selection. We applied the normal distribution’s right-tail probability density function (PDF) to infer the *p*-values as provided in R (R Core Team, 2020).

We used–weir-fst-pop in vcftools (Danecek et al., 2011) based on Fst estimation (Weir and Cockerham, 1984) to analyze selection signature between the population of each taurine breeds (Fleckvieh, Kazakh, Mongolian, and Yanbian) against a combination of all indicine and admixed Chinese individuals. Fst values were averaged with 10 Kb non-overlapping windows. The probability density function of normal distribution inferred the *p*-values considering the right tail only. Genome-wide significance -log_10_(p) of 7.301 was set as the threshold. For all the analysis, manhattan plots were built using the qqman R-package (Turner, 2018).

### Functional annotation and gene expression

SnpEff (Cingolani et al., 2012) annotated SNVs and windows positions above the threshold using Ensembl version of ARS_UCD1.2 annotation file. For functions of individual genes, we referred to the one listed in https://www.genecards.org/and https://www.ncbi.nlm.nih.gov/gene/. We further considered only genes indicated by SNVs within coding regions. We used pantherdb.org to classify the functionality of associate genes listed by different statistical methods to its gene ontology (GO) terms. We annotated significant genes indicated by the tests for their expression level using cattle gene atlas (Fang et al., 2020). We retained the information of maximum expression level in fragments per kilo base per million mapped reads (FPKM) and its corresponding tissue where the maximum FPKM is found for each indicated gene. In this repository, the mean and standard deviation for FPKM across 91 tissues and 447 individuals are 26.79 and 730.56, respectively.

## Results

**FIGURE 1:**
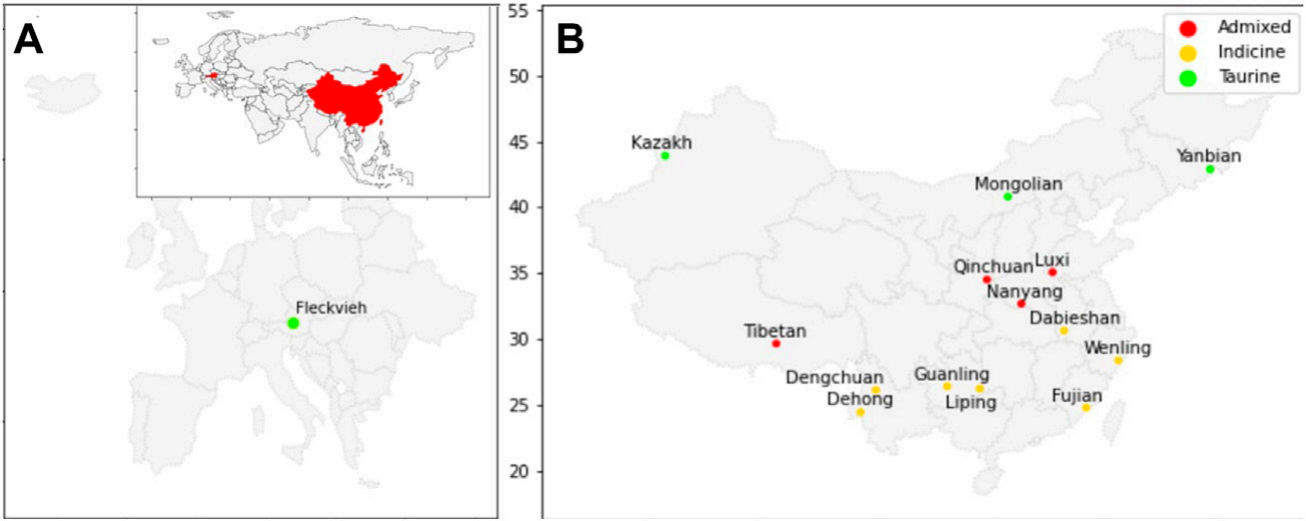
Map of origin for cattle used in this study; **(A)** Austrian Fleckvieh origin on European map with an inset of China and Austria in the world map; **(B)** Position of Chinese cattle breeds on the maps.

Whole-genome sequence data of 60 individuals from 14 breeds of Chinese cattle and Austrian Fleckvieh were aligned against the ARS_UCD1.2 reference genome. On average, there were ∼316 million paired reads per individual FASTQ, with length varies from 90 to 148 bases per read. In total, ~60 million SNVs passed the set of hard filtration for all autosomes with an average depth of ∼9 × Table 1 indicated alignments summary for each breed with details provided in Supplementary Table S1. Figure 1 depicted the origin of Chinese cattle and Austrian Fleckvieh on the world map.

**TABLE 1:** Alignment summary of the dataset.

Breeds	Species^a^	N animals	Total reads in million^b^	Read length^c^	Mapped reads^d^	Depth^b^
Fleckvieh	*B. taurus*	10	326.52	101	0.977	5.823
Kazakh	*B. taurus*	6	250.32	131	0.998	8.859
Mongolian	*B. taurus*	12	210.78	139	0.998	8.259
Yanbian	*B. taurus*	10	226.90	137	0.998	8.513
Dabieshan	*B. indicus*	2	345.20	96	0.992	10.257
Dehong	*B. indicus*	2	342.82	93	0.997	10.052
Dengchuan	*B. indicus*	2	346.43	94	0.997	10.346
Fujian	*B. indicus*	2	332.11	95	0.997	9.988
Guanling	*B. indicus*	2	340.23	96	0.997	10.269
Liping	*B. indicus*	2	338.78	96	0.997	10.097
Wenling	*B. indicus*	2	339.14	90	0.997	9.387
Luxi	*Admixed*	2	324.72	95	0.998	10.133
Nanyang	*Admixed*	2	369.14	96	0.998	10.333
Tibetan	*Admixed*	2	301.14	96	0.998	9.636
Qinchuan	*Admixed*	2	347.76	93	0.998	9.846

a Assigned species were based on the principal component analysis carried out in this study—Admixed of *B* *taurus* and *B. indicus*; Superscript b, c, d, e were the average values from individuals in each respective breed.

b Depth values inferred from SNVs, in the final VCF, file.

### Principal component and admixture analysis

**FIGURE 2:**
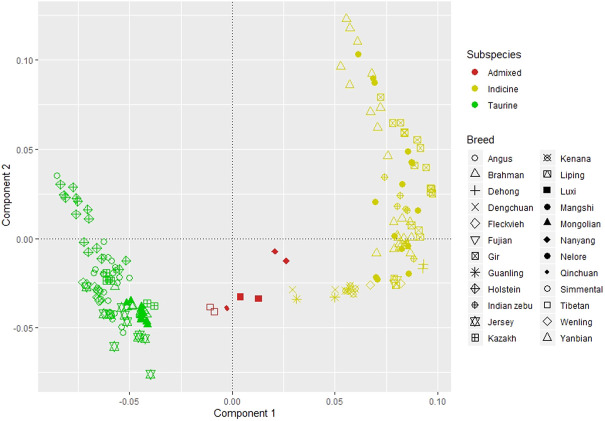
Principal component analysis; component 1 explains 47.37 percent of variants and component 2 for 10.20 percent.

We inferred the population structure using PCA from ∼4.5 million SNVs shared by all individuals in the dataset. The PCA explains 47.37, 10.20, 6.68, 6.52, and 5.89 percent of variance for components one to five, respectively. Figure 2 depicted the clustering of all individuals based on the first and second components. We observed a clear separation between the taurine and indicine cattle by the first component regardless of its origin. The three Chinese breeds, Kazakh, Mongolian, and Yanbian, were clustered together with Austrian Fleckvieh and other renowned *B. taurus* breeds, such as Angus, Holstein, and Jersey. Nanyang, Luxi, Qinchuan, and Tibetan were admixed as they were between clusters of *B. taurus* and *B. indicus*. While Dabieshan, Dehong, Dengchuan, Fujian, Guanling, Liping, and Wenling were clustered together with other *B. indicus* breeds such as Brahman, Nelore, and Gir.

**FIGURE 3:**
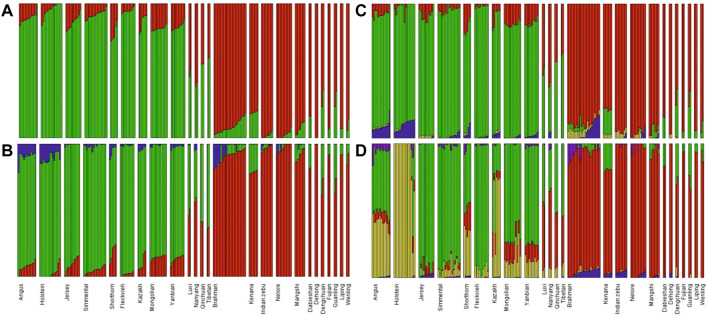
Admixture analysis using **(A)** K = 22; **(B)** K = 3; **(C)** K = 4; and **(D)** K = 5.

Admixture analysis for those breeds using K from two to five supported the results of PCA, see Figure 3. Thus, based on these results and considering the number of individuals in each breed, we pooled individuals into separate groups for further selection signature analysis, i.e. Fleckvieh, Kazakh, Mongolian, Yanbian, CHBI_Low (seven Chinese *B. indicus* breeds), CHBI_Med (four Chinese admixed breeds), and CHBI(combination of CHBI_Low and CHBI_Med).

The PCA and admixture results matched the geographical origin of individuals as in Figure 1. Kazakh, Mongolian, and Yanbian were sampled from the northern part of Chinese. CHBI_Med individuals were from the middle latitude of the country. While the CHBI_Low individuals were originated from the southern part of the country. Coincidentally, Austrian Fleckvieh originated from a region with latitude around 48°, while three Chinese taurine breeds were also coming from a similar temperate climate of 42° latitudes.

### Comparison of methods in scanning for positive selection

**FIGURE 4:**
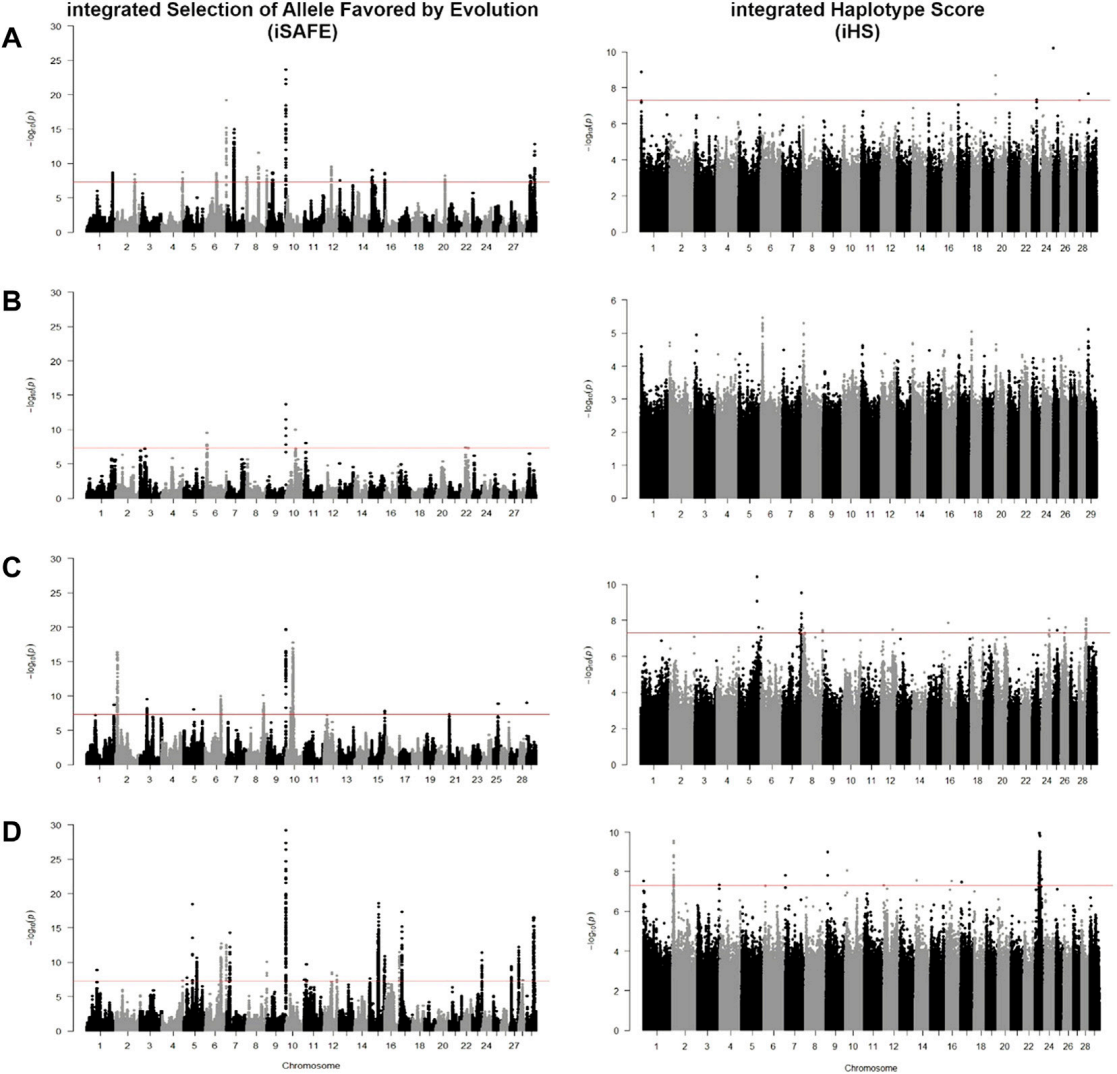
Manhattan plot for iSAFE and iHS tests for **(A)** Fleckvieh; **(B)** Kazakh; **(C)** Mongolian; and **(D)** Yanbian.

**FIGURE 5:**
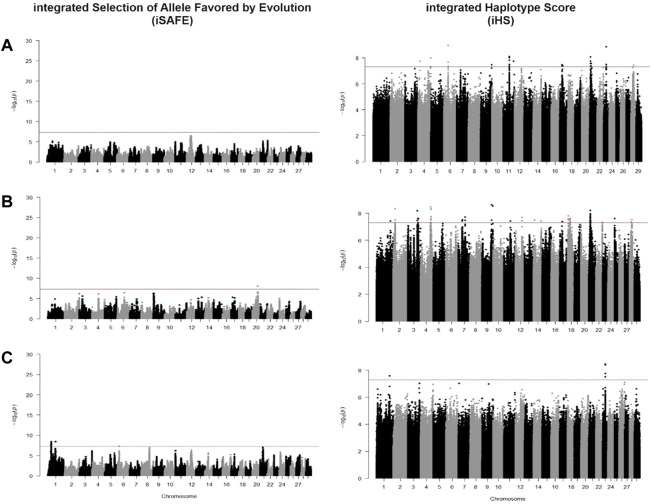
Manhattan plot for iSAFE and iHS tests for **(A)** CHBI; **(B)** CHBI_Med; and **(C)** CHBI_Low.

We identified candidate SNVs for positive selection using two within-population tests of iSAFE and iHS. Additionally, we performed one cross-population test of Fst between taurine breeds against CHBI individuals. Phased vcf files were separated for each pool of individuals and underwent both tests, respectively. Table 2 indicated the descriptive statistics of significant SNVs of respective tests and individual pools. Figure 4 and Figure 5 depicted the manhattan plots of *B. taurus* and other CHBI groups, respectively.

**TABLE 2:** Summary of output scores of SNVs and windows from iHS, iSAFE, and Fst tests.

Pools^a^	iSAFE test				iHS test				Fst test^f^			
	Mean ± SD^b^	Sign. SNVs^c^	Intergenic^d^	Gene^e^	Mean ± SD^b^	Sign. SNVs^c^	Intergenic^d^	Gene^e^	Mean ± SD^g^	Sign. Windows^h^	Intergenic^i^	Gene^j^
Fleckvieh	0.07 ± 0.05	2,264	1,764	161	-0.01 ± 0.70	6	3	2	0.08 ± 0.05	301	177	167
Kazakh	0.10 ± 0.08	1,502	1,039	56	-0.53 ± 1.07	0	0	0	0.06 ± 0.05	336	190	185
Mongolian	0.06 ± 0.04	3,446	2,656	111	-0.15 ± 0.64	41	18	7	0.08 ± 0.05	334	185	179
Yanbian	0.05 ± 0.03	5,068	3,681	258	-0.09 ± 0.65	91	36	8	0.07 ± 0.05	334	188	182
CHBI	0.11 ± 0.07	0	0	0	-0.01 ± 0.60	30	23	4	NA	NA	NA	NA
CHBI_Med	0.10 ± 0.07	469	368	3	-0.49 ± 0.75	59	43	11	NA	NA	NA	NA
CHBI_Low	0.09 ± 0.06	1,648	881	28	0.02 ± 0.70	5	4	1	NA	NA	NA	NA

a Pools: grouping of individuals - first four are specific *B. taurus*, CHBI_Low (seven Chinese *B. indicus*), CHBI_Med (four Chinese admixed), and CHBI (combination of CHBI_Low and CHBI_Med).

b Mean and SD, of raw values for SNVs, reported in each respective test.

c Number of SNVs, passing the threshold of genome-wide significance (-log_10_(p) = 7.301).

d Number of SNVs, annotated to intergenic regions.

e Number of SNVs, annotated to coding regions of genes.

f Fst test of respective breed against CHBI.

g Mean and SD, of Fst values for all windows.

h Number of windows passing the threshold of genome-wide significance (-log_10_(p) = 7.301).

i Number of windows annotated to intergenic regions.

j Number of windows annotated to coding regions of gene.

Using iSAFE, we found several peaks of SNVs for *B. taurus* breeds. For all *B. taurus*, the strongest signals come from chromosome nine around 104.4 Mb. CHBI_Med and CHBI_Low had independently signals on chromosome 20 and 1, respectively. We did not find significant SNVs for CHBI. Around 71 percent of SNVs indicated as significant in the iSAFE test were annotated to intergenic regions, as stated in Table 2. A list of significant genes indicated by iSAFE, iHS, and Fst is provided in Supplementary Tables S2-S4.

In Fleckvieh, Kazakh, Mongolian, and Yanbian, two genes of *ENSBTAG00000045624* and *ENSBTAG00000047934*, known also as *OR10D1M*, were genes indicated by the most significant iSAFE score. The later gene belongs to the olfactory receptor family, which interacts with odorant molecules in the nose, initiating the neuronal response that starts a sense of smell. This gene was neither found in significant regions of CHBI_Low nor CHBI_Med. *ENSBTAG00000053225* (*OR8B60*) and *ENSBTAG00000050546* (*OR8AR1*) were olfactory receptor genes indicated in Fleckvieh, Kazakh, and Yanbian. All these top indicated genes are located at chr 9, around 104.3 Mb. Within 100 Kb vicinity of these olfactory genes, we found *FAM120B, DLL1, PSMB1*, and *PDCD2. FAM120B* has several associations of twinning rate in mammals, fat deposition in chicken and inflects pig sperm maturation during spermatogenesis due to its function in adipogenesis regulation of *PPARG* (Vinet et al., 2012; Moreira et al., 2015; Gòdia et al., 2020). In human, *DLL1* plays role in Notch signaling pathway regulating cell differentiation and proliferation in embryonic development and maintenance of adult stem cells (Jaleco et al., 2001). In cattle, activation of Notch pathway by miRNA targeting *DLL1* leads to restrain adipose differentiation which might lead to different subcutaneous adipose tissue between Wagyu and Holstein (Guo et al., 2017). While in embryo development, *in vitro* expression of *PSMB1* is significantly reduced after bovine oocyte maturation (Adona et al., 2011). Similarly, *PDCD2* plays also role in embryo development as indicated of its activation during bovine 16-cell stage (Graf et al., 2014).

Overlapped genes found in Fleckvieh and Yanbian were *ACP1, ALKAL2, ENSBTAG00000045328, ENSBTAG00000045624, ENSBTAG00000047934, ENSBTAG00000050546, ENSBTAG00000051204, ENSBTAG00000053225, POLN, SH3YL1* and *U6*. *ALKAL2* is associated with reproduction function and upregulated in granulosa cell of bacteria-infected uterus in Holstein heifers (Horlock et al., 2020) while *POLN* was reported to influence mature body size in US sheep population (Posbergh and Huson, 2021). For CHBI_Med, *CDH12* is associated with longevity and desaturation of milk fatty acids as reported in few dairy cattle (Mészáros et al., 2014; Cecchinato et al., 2019). For CHBI_Low, *CYP2U1* is linked to milk fat secretion in Sahiwal cattle in India (Illa et al., 2021).

Using iHS, we did not find significant SNVs for Kazakh. In contrary, Yanbian had 91 significant SNVs. These SNVs were observed as a peak in chromosome 23. Significant SNV at chr23:26, 067, 413 was detected both in Yanbian and Fleckvieh. For Mongolian, we observed several peaks on chromosomes five and 7. A total of 30, 59, and five SNVs were above the threshold for CHBI, CHBI_Med, and CHBI_Low, respectively, with no overlaps among them. For all groups, the mean iHS score was generally in a negative value except for the CHBI_Low. 58 percent of significant SNVs in iHS were annotated to intergenic regions, as indicated in Table 2.

For Fleckvieh, SNVs with significant iHS at chromosome 20 around 3.8 Mb overlapped to *STK10* gene, which is significantly associated with slaughter weight and carcass quality in several beef cattle breeds (Karisa et al., 2013; Hay and Roberts, 2018). For Mongolian, SNVs with significant iHS scores were overlapped with the novel gene of *ENSBTAG00000050324, PTPRM, GRID1, CACNA1C, SORCS3, NRG3*, and *TXNDC2. PTPRM* has extended function in regulating cellular growth, differentiation, mitotic cycle, and is associated with scrotal circumference in Nellore and Brahman cattle (Melo et al., 2019). *GRID1* is known for its function in the central nervous system and is down-regulated in fetuses carrying deletion variants in *PEG3* domain leading to stillbirth (Flisikowski et al., 2012). *CACNA1C* is linked to immune defense and was hyper-methylated in Angus during stress of high-temperature high-humidity period (Del Corvo et al., 2021). *SORCS3* was highly associated with temperament trait and average daily gain (Xu et al., 2019; Shen et al., 2022). *NRG3* is associated with fat yield component in sheep production (García-Gámez et al., 2012). While *TXNDC2* is linked to average daily gain and age at puberty in Korean cattle (Edea et al., 2020).

For Yanbian, the top genes indicated by significant iHS score were *ENSBTAG00000026163, ENSBTAG00000007075, C2H2orf88, TBCA, HIBCH, TMEM71, SMYD3, ARFIP1,* and *HDAC4*, which all these genes play a role in cellular proliferation and transcription factors. *TBCA* is associated with sire conception rate in the US Jersey cattle (Rezende et al., 2018). *HIBCH* is one of candidate genes in association study of calving performance in Charolais population (Purfield et al., 2015). While *ARFIP1* is associated with milk production traits in Holstein (Lee et al., 2016).

For CHBI, significant SNVs were in novel genes of *ENSBTAG00000026163, ENSBTAG00000053922, PBLD*, and *AFDN*, which encodes a multi-domain protein involved in signaling and organization of cell junctions during embryogenesis. For CHBI_Med, *ENSBTAG00000020723, PDE10A, AKAP13, KLHL25, ENSBTAG00000054043, FAM234A, PODXL, RUVBL1*, and *ABHD1* were indicated. *AKAP13* and *KLHL25* were also reported as selection candidates in North African cattle (Ben-Jemaa et al., 2020). *RUVBL1* is associated with tolerance of African cattle towards heat and parasites stress (Taye et al., 2017a; Yougbaré et al., 2021). While in CHBI_Low, *ENSBTAG00000026163* is a gene indicated by significant SNV.

**FIGURE 6:**
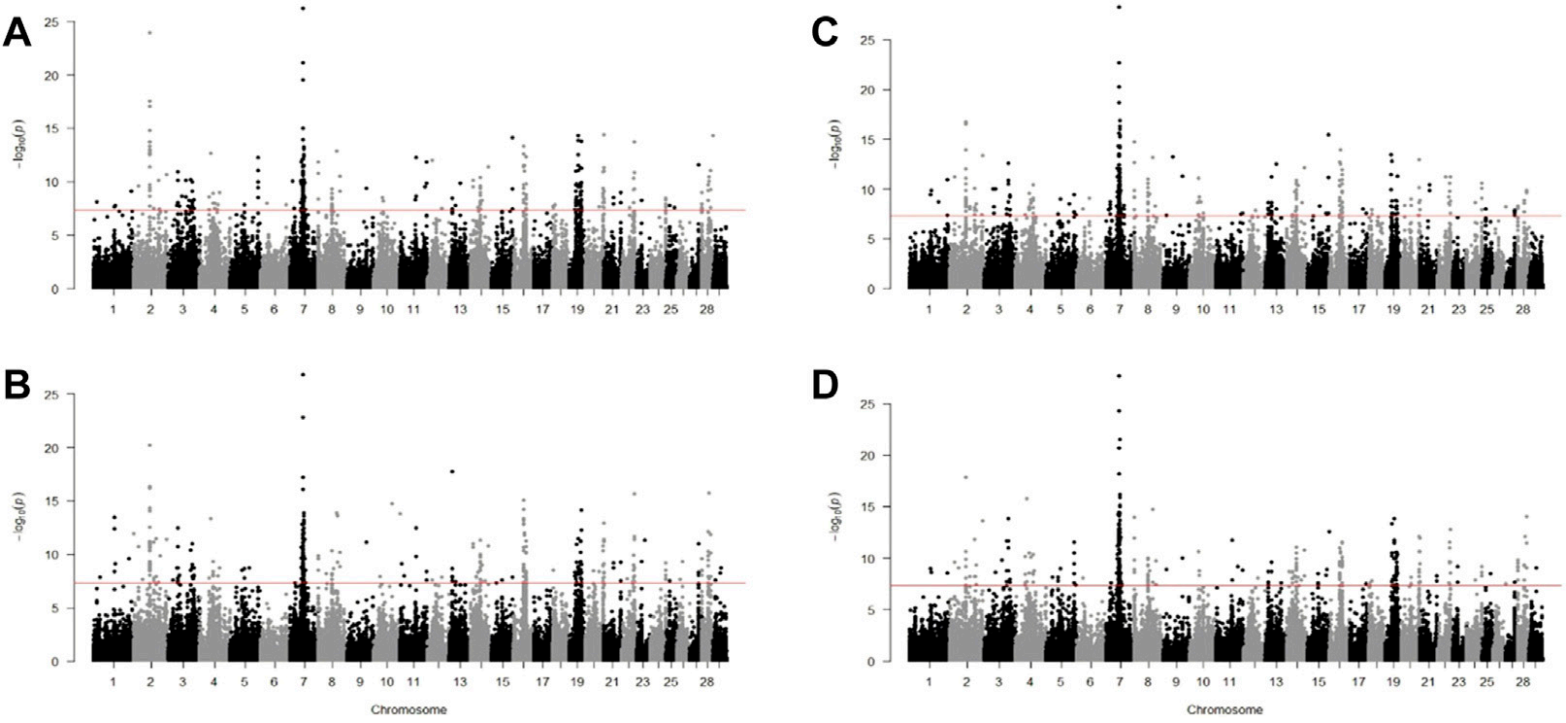
Manhattan plot for Fst test for **(A)** Fleckvieh; **(B)** Kazakh; **(C)** Mongolian; and **(D)** Yanbian against CHBI.

Using Fst test, we found a similar pattern among the *B. taurus* cattle as shown in Figure 6. There were 224 similar significant windows in Fleckvieh Kazakh, 181 in Fleckvieh-Mongolian, 208 in Fleckvieh-Yanbian, 185 in Kazakh-Mongolian, 217 in Kazakh-Yanbian and 222 in Mongolian-Yanbian. Among these, 132 windows were significant in all of Fleckvieh, Kazakh, Mongolian, and Yanbian. Out of 132, 73 windows were annotated to intergenic regions and the rest to 60 genes. Within these genes, *LCT* was reported as selection candidates in several Italian cattle and its mutations in humans, irrespective of location and mutation type, are linked to congenital lactase deficiency (Torniainen et al., 2009; Sorbolini et al., 2016). *DHRS3* is known for its importance in retinoic acid metabolism and is essential in regulating body axis formation during embryonic development (Kam et al., 2013). *PRKCZ*, where young calves are exposed to hypoxia, leads to anti-replication activity of cells related to this gene in pulmonary artery adventitia (Das et al., 2008).

Several genes indicated by Fst were found exclusively in three Chinese taurine breeds and not in Fleckvieh. These genes might be related to the adaptation process to the local habitat. For example, *ANXA10* was detected as a selection candidate in Kholmogory cattle and deletion-type CNV of 34 kb identified in this gene was linked to embryonic mortality in Japanese Black cattle (Sasaki et al., 2016; Yurchenko et al., 2018). *C14H8orf34* is associated with claims on epinephrine hormone excretion in the urinary due to many pathways of metabolism acceleration under stress situations (de Camargo et al., 2015). *CACNA2D3* plays role in active calcium ion transport and was highly expressed in *Longissimus Lumborom* than *Psoas Major* muscles after postmortem in Chinese Jinjiang cattle (Yu et al., 2019). *PAX5* is associated with the proportion of black color in Holstein (Hayes et al., 2010). *PLAG1* is a known gene with pleiotropic effects on body weight and fertility traits (Fortes et al., 2013; de Camargo et al., 2015; Yurchenko et al., 2018). *TAC3* was associated with reproduction process as highly expressed in non-pregnant heifers compared to heifers that later became pregnant (Dickinson et al., 2018).

### Go classification and expression level

GO classification for genes indicated by iHS, iSAFE, and Fst tests is provided in Supplementary Table S5. Cellular process (GO:0009987) was the top GO term indicated by significant genes irrespective of the test and individuals pool. Metabolic process (GO:0008152) was the second top GO term for iSAFE test in Fleckvieh and Mongolian, while for Kazakh and Yanbian, the second top was biological regulation (GO:0065007). There were 10, 5, 2, and 13 genes for developmental process (GO:0032502) indicated by iSAFE test for Fleckvieh, Kazakh, Mongolian, and Yanbian, respectively. In general, Yanbian had more coverage to broader GO terms like reproduction (GO:0000003), reproductive process (GO:0022414), multi-organism process (GO:0051704), growth (GO:0040007) perhaps due to the higher number of genes detected by iSAFE compared to Fleckvieh, Kazakh, and Mongolian.

**FIGURE 7:**
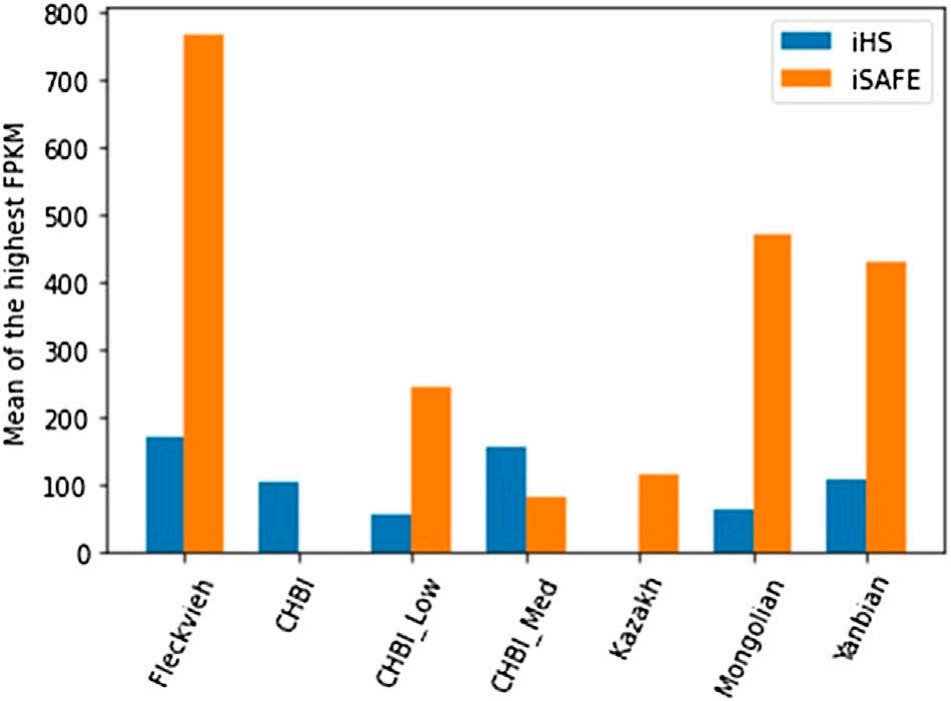
Mean of the highest FPKM for all significant genes indicated by iHS and iSAFE tests in each respective individual pool.

We annotated significant genes indicated by iHS and iSAFE toward their maximum expression level (FPKM) and the corresponding tissue as listed in the repository of cattle gene atlas (Fang et al., 2020). Rectangular bar in Figure 7 depicted the average FPKM of significant genes for the corresponding test and individual pool. In general, the mean FPKM values by both tests were higher than the mean of FPKM records of the full repository (26.79). iSAFE indicated higher mean of FPKM than the iHS except for CHBI_Med and CHBI where no SNVs were significant in iSAFE tests. Supplementary Figures S1, S2 depicted cloud plots for the associated tissues with the FPKM for iSAFE and iHS tests. Genes indicated by iHS were mostly highly expressed in the ileum tissue as indicated in Fleckvieh, Yanbian, CHBI, and CHBI_Low. For iSAFE, the significant genes for all individual pools were all highly expressed in the sperm, see Supplementary Figure S1.

For Fleckvieh, *TFF1* was the gene listed by iSAFE with the highest FPKM (10,413) in abomasum tissue. *TFF1* was reported to be associated with mammary development and secretion of minerals to the bovine milk (Stella et al., 2010; Gao et al., 2017). Ten modifiers and one low impact were estimated for the SNVs indicated within *TFF1* in Fleckvieh. For Kazakh, the highest expressed gene was *ALDH1A2* with 250 FPKM from stalk median eminence tissue and is associated with carcass weight in beef cattle (Willing et al., 2012). Functional modifiers’ impact were annotated for all 13 SNVs in *ALDH1A2*. For Mongolian cattle, *ALDOA* was the highest expressed gene with 12,960 FPKM in chorid plexus tissue in the brain. *ALDOA* is primarily related to glycolytic and energy metabolism (Wærp et al., 2019). *TNNT2* identified in Yanbian was highly expressed in heart tissue with 5006 FPKM. Seven SNVs with modifier impact were associated with this gene, which is related to the striated muscle contraction due to intracellular calcium ion concentration and found in a previous study as selection candidates in Holstein cattle (Taye et al., 2017b).

## Discussion

This study indicated a gradual shifting of taurine cattle in northern China to admixed and pure indicine cattle towards the southern part of China, similar to the report from the previous studies (Chen et al., 2018; Zhang et al., 2020). Zhang et Al (2020) indicated that Mongolian and Kazakh, two Chinese taurine in our study, were well adapted to cold winters. They suggested that the admixture and introgression of taurine and indicine from north to south can be affiliated to loci in the genome, which might help individuals adapt to the local environment (Wu et al., 2018). A previous study (Gao et al., 2017) suggested that Chinese taurine cattle in the north shared the same genetic ancestry to several Central Asia, Russian-Yakutstk, Korean and Japanese cattle (Turano-Mongolian) in the greater region due to past activities of nomads and the Mongolian empire.

Previous studies used various selection signature methods and normalized the outputs score using specific windows, in basepairs or based on the number of SNPs, to identify the candidate regions (Qanbari et al., 2014; Xu et al., 2015; Yurchenko et al., 2018; Bhati et al., 2020). We applied a similar approach for the Fst test using non-overlapping 10 Kb windows. However, for within-population tests of iHS and iSAFE, we did an experimental analysis to point out the causal SNV mutations in coding regions that significantly drive selective sweeps in genome-wide level. We found that iHS indicated fewer signals passing the genome-wide significant threshold than iSAFE in any breed. This is in line with simulations in the original paper where iSAFE could detect almost double the signals for favoured mutations than iHS (Akbari et al., 2018). Both methods were associated with declining performance in detecting mutations in regions that are closed to fixation, yet we found no overlapped genes indicated by these two tests.

Generally, our study suggested higher selection signals for taurine than indicine cattle in both iSAFE and iHS tests. For example, in the iHS test, Yanbian had 91 significant SNVs while CHBI_Med had only 59. Similarly, in the iSAFE test, Yanbian had around five thousand significant SNVs while CHBI_Med had a far less, around 469 SNVs. Our finding is similar to previous study where indicine cattle of Gyr and Nelore had substantially fewer regions proposed as selection evidence compared to taurine cattle of Brown Swiss and Angus (Utsunomiya et al., 2013). Moreover, pools of indicine cattle in our study were a combination of several breeds due to limited number of individuals to a suggested minimum of six individuals for better population genetic analysis (Willing et al., 2012). Thus as the results, we observed decayed of the iSAFE signal in CHBI as the combination of CHBI_Low and CHBI_Med, compared to the scenario when both groups were tested independently. We assumed that the signals for each indicine breed would be more significant and apparent if the sample size were equal to the taurine breed. However, due to the circumstances, we could not do it for the current study.

As indicated in the results section, more than half of the signals fall under intergenic regions. We did not consider SNVs found in those regions and retained only SNPs in the coding regions. Within these SNVs, several were without official gene ID names. For example, 15 SNVs creating a peak in iHS test chromosome 23 of Yanbian were in an active transcription of ENSBTAG00000007075 gene. According to https://bgee.org/?page=gene&gene_id=ENSBTAG00000007075, this gene was described as a major histocompatibility complex, class I, A-like precursor and has paralogs to BOLA-A and JSP.1 genes. And has an association with feeding efficiency in Norwegian Red heifers where it is upregulated during diet changes from low-protein-high-energy to low-protein-low-energy feed (Wærp et al., 2019). Yet, for GO classification, we considered only genes with official ID names overlooking functions of genes with prefix ENBST names.

*KIT* was indicated as one of selection candidate genes affecting coat colors (Flori et al., 2009; Stella et al., 2010; Xu et al., 2015). In chromosome six around 70 Mb, where KIT is located, there were 236 SNPs in the phased genotypes. Yet, we did not find any significant SNVs passing the genome-wide threshold, though the maximum iSAFE scores ranges from 0.04 to 0.20 among the *B. taurus,* see Supplementary Figure S3 *.* Apparently, the threshold for iSAFE on genome-wide level has biased the findings to SNVs within highly-scores segments. Meanwhile each genome segment may have different significance level for assigning SNV as the best candidate of selection. This was demonstrated in the original manuscript where a SNV with score of 0.10 was the best candidate in *HBB* while score of 0.61 was the best candidate for *EDAR* (Akbari et al., 2018). However, genes indicated by genome-wide threshold of iSAFE might act as the driver of selection within the LD segments as they had the highest scores. Though the functionality of these genes were quite spurious, generally they had higher expression in tissues, particularly in sperm, compared to ones indicated by iHS.

In the Fst test, we found *PAX5* as a candidate gene in three Chinese taurine breeds, not shared with Fleckvieh, which function is associated with black color patterns (Hayes et al., 2010). In general, Chinese indigenous cattle, including these three breeds, are considered as ‘yellow’ cattle, thought the they are actually in different level of brownish colors. A specific *PAX5* might affect the color pattern of these breeds, separating them from other Turano-Mongolian cattle, such as the Mongolian and Korean cattle, which still retain their original dark-brown coat color pattern (Gao et al., 2017).

Our findings suggested that three Chinese taurine cattle breeds shared a considerable amount of candidate regions with Fleckvieh. Though we can confirm that there was no recorded genetic material exchange between Austria and China, it was reported that there were programs for improving the productivity of local breeds by crossing to European breeds in the last decades (Gao et al., 2017). As those European breeds might have similar characteristics as Fleckvieh, thus we cannot attribute similarity between Austrian Fleckvieh and Chinese taurine solely due to independent co-selection of nature, but also possibly due to recent crossing with other European breeds.

## Conclusion

Our study confirmed a gradient of taurine and indicine admixed cattle from north to south of China. More significant SNVs were identified using iSAFE than the iHS for the candidate of positive selection and more detectable signals in taurine than in indicine individuals. However, combining individuals of different breeds decaying the iSAFE signals. From both tests, significant SNVs are linked to the olfactory receptors, production, reproduction, and temperament traits in taurine cattle, while heat and parasites tolerance in the admixed individuals. Fst test suggests similar patterns of population differentiation between Fleckvieh and three Chinese taurine breeds against Chinese indicine breeds. However, there are genes shared only among the Chinese taurine, such as PAX5, affecting black coat color, which might underlying differences of these breeds to other Turano-Mongolian cattle.

## Data Availability

The datasets presented in this article are not readily available because the animals and subsequently their genomic data are property of respective breeding organizations. Requests to access these datasets should be directed to GM (gabor.meszaros@boku.ac.at) for the data set from Austria or to XD (xiangdongding@hotmail.com) for the data sets from China.
