# Supplementary material for: Favored single nucleotide variants identified using whole genome Re-sequencing of Austrian and Chinese cattle breeds
Source: Front Genet. 2022 Sep 27;13:974787. doi: 10.3389/fgene.2022.974787 (PMC9552183; doi:10.3389/fgene.2022.974787)
Supplement: Supplementary file 1 [file DataSheet2.docx]

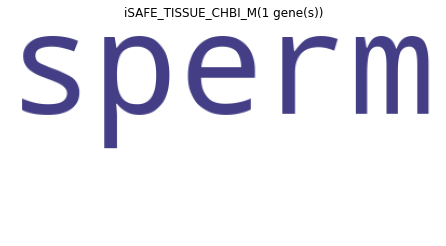

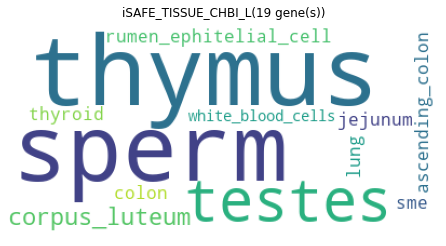

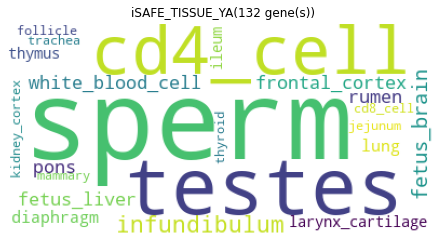

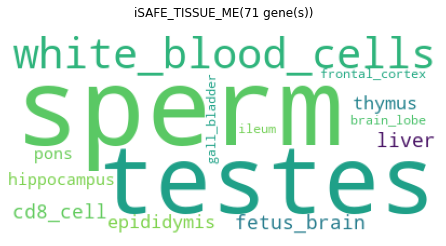

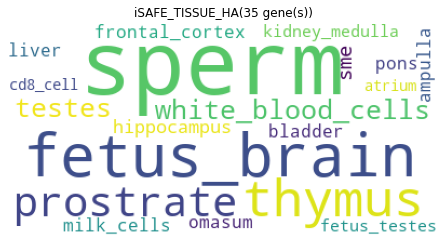

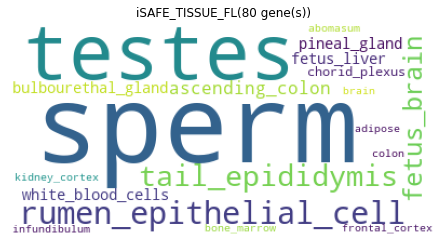
Supplementary figure 1. Cloud plot of tissues with the highest FPKM for respective significant genes in iSAFE test for individual pool of : a) Fleckvieh, b) Kazakh, c) Mongolian, d) Yanbian, e) CHBI_Med, f) CHBI_Low

f)

e)

d)

c)

b)

a)


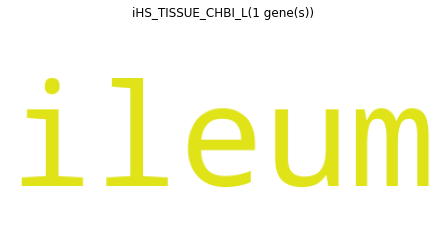

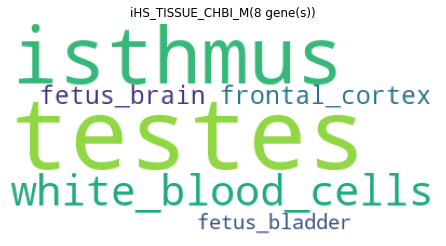

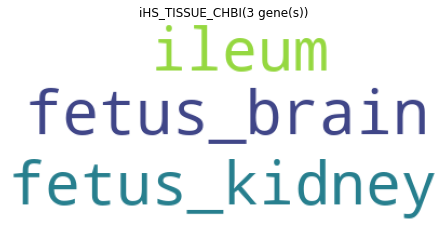

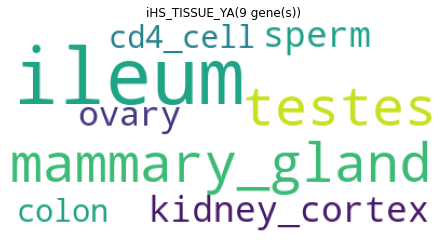

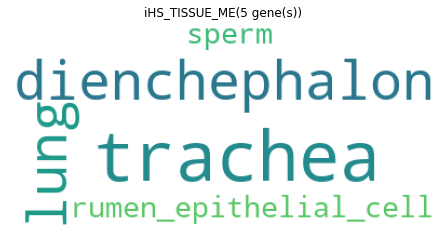

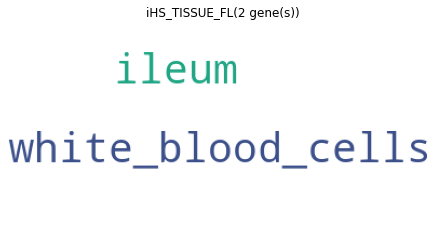
Supplementary figure 2. Cloud plot of tissues with the highest FPKM for respective significant genes in iHS test for individual pool of : a) Fleckvieh, b) Mongolian, c) Yanbian, d) CHBI, e) CHBI_Med, f) CHBI_Low

f)

e)

d)

c)

b)

a)


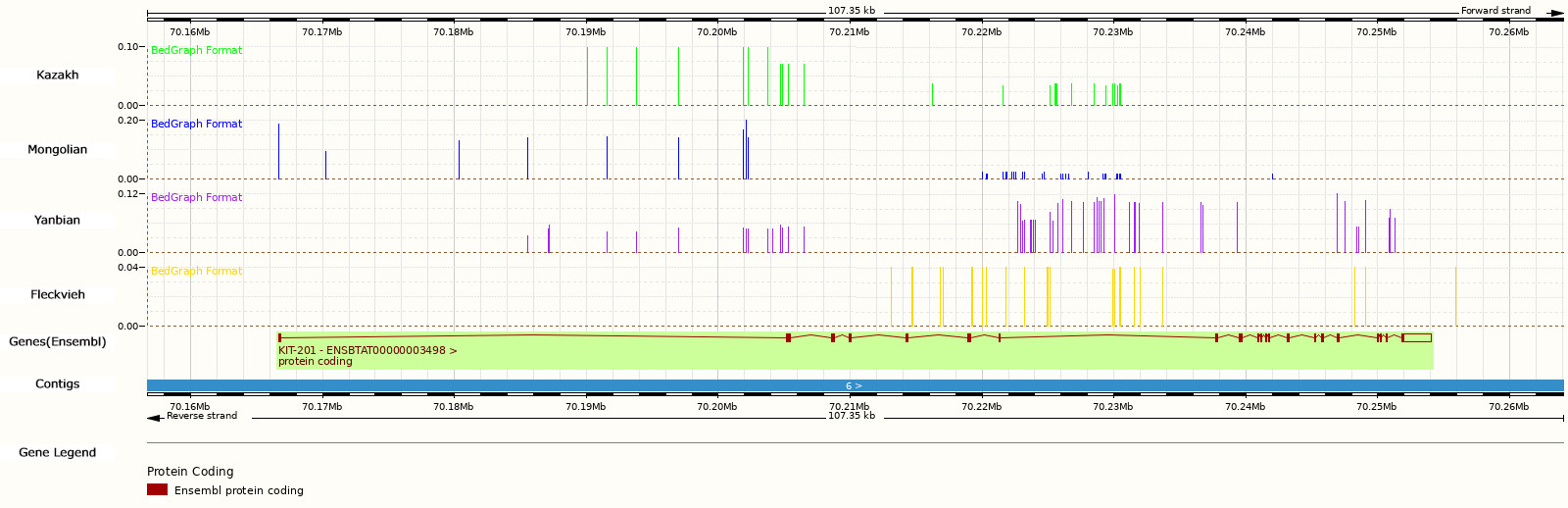


Supplementary figure 3. Track panels show iSAFE scores around *KIT* gene for Kazakh, Mongolian, Yanbian, and Fleckvieh
